# The impact of pathogen reduction on ABO isoagglutinin titers in apheresis platelets

**DOI:** 10.1016/j.htct.2025.103840

**Published:** 2025-05-23

**Authors:** Mikayel Yeghiazaryan, Yembur Ahmad, Jessie Singer, Vaanush Nazaryan, Craig Fletcher, Yamac Akgun

**Affiliations:** a Department of Pathology and Laboratory Medicine, Children’s Hospital Los Angeles, Los Angeles, CA, USA; b Department of Pathology, Keck School of Medicine of USC, Los Angeles, California, USA

**Keywords:** Pathogen reduction, Apheresis platelets, Isoagglutinin titers, Solid phase

## Abstract

**Background:**

Platelet transfusions are a cornerstone of modern medical care, used across various clinical contexts. Ensuring the compatibility of blood products, especially regarding ABO isoagglutinins, is critical to minimize adverse reactions. Pathogen reduction technologies have been widely adopted to enhance the safety of blood products, however, the impact of such treatments on ABO isoagglutinin titers in platelet products remains unclear.

**Methods:**

This study analyzed 60 apheresis platelet donations, including type O, A, and B donors, using the INTERCEPT® Blood System for pathogen reduction. Samples were collected both from donor whole blood at the time of apheresis (Retention) and from the final pathogen-reduced platelet product after it had passed through the compound adsorption device (Post-CAD). ABO isoagglutinin titers, including both IgM and IgG classes, were measured using solid-phase technology on the NEO Iris platform.

**Results:**

This study found a significant reduction in IgM isoagglutinin titers in Post-CAD samples, with 99 % of Retention titers being greater than or equal to their Post-CAD counterparts. IgG titers exhibited more variability, with 9 % of Post-CAD samples displaying higher titers than Retention samples. Statistical analysis confirmed differences between Retention and Post-CAD samples for both IgM and IgG titers, with p-values <0.05 in most comparisons.

**Conclusion:**

Pathogen reduction using the INTERCEPT® Blood System effectively reduces ABO isoagglutinin titers in apheresis platelets, potentially lowering the risk of hemolytic transfusion reactions. This reduction is beneficial for safer out-of-group platelet transfusions, especially in vulnerable populations such as pediatric patients. These findings support the continued use of pathogen-reduced platelets in transfusion medicine to enhance both safety and availability of blood products.

## Introduction

Platelet transfusions are a vital component of modern medical care, utilized across various clinical settings ranging from trauma and surgery to the management of hematological disorders. The compatibility of blood products, including platelets, is paramount to ensure patient safety and the efficacy of treatment [1]. The ABO blood group system, characterized by the presence or absence of A and B antigens on red blood cells, plays a central role in blood transfusion compatibility [2]. In addition to these antigens, individuals also possess naturally occurring antibodies known as isoagglutinins, which are directed against the ABO antigens absent from their own blood [3]. ABO antibodies develop in individuals at 3–6 months of age and reach adult levels at 5–10 years [4]. They are primarily IgM, and IgG antibodies often belonging to the IgG_2_ subclass. Historically, ABO matching has been considered less critical for platelet transfusions compared to red blood cell transfusions due to the lower expression of ABO antigens on platelets and the shorter lifespan in circulation [5]. As a result, out-of-group platelet transfusions, where the donor and recipient have different ABO blood groups, have been commonly practiced, especially in situations where ABO-matched platelets are unavailable or in high demand [6].

Despite the prevailing acceptance of out-of-group platelet transfusions, concerns regarding the potential risks associated with ABO-incompatible platelet transfusions have prompted a reevaluation of transfusion practices [7]. One significant development in this regard is the recognition of ABO titers as a valuable tool in assessing the suitability of out-of-group platelet transfusions [8]. The semi-quantitative assessment of ABO isoagglutinin titers is valuable for evaluating the compatibility of blood products with the recipient and for minimizing the risk of adverse reactions such as hemolytic transfusion reactions [9]. Traditional methods for titration involve labor-intensive techniques such as tube agglutination or gel centrifugation. However, advancements in technology have introduced automated methodologies, offering high-throughput and standardized approaches for ABO isoagglutinin titration [10].

In addition, pathogen reduction of platelets represents an important advancement in blood safety technology and is increasingly being adopted by blood centers and hospitals worldwide [11]. Psoralen is a photosensitive compound that, when activated by ultraviolet (UV) light, forms cross-links between nucleic acids, thereby preventing replication and transcription of DNA and RNA in pathogens such as bacteria, viruses, and parasites [12]. Residual psoralen and byproducts are removed by adsorption via the compound adsorption device (CAD) to reduce toxicity [13].

Psoralen treatment of platelets is an effective method of pathogen reduction, yet its impact on the levels of ABO isoagglutinins in the plasma of the final treated product is unknown. This study aims to evaluate ABO isoagglutinin titers (both IgM and IgG) in platelet donations using automated solid-phase technology on the NEO Iris platform (Werfen, previously Immucor, Inc). By comparing titers between donor whole blood samples collected at the time of apheresis and final pathogen-reduced platelet product samples, this study seeks to identify any changes in isoagglutinin levels that may occur during the manufacturing process.

## Material and methods

Sixty apheresis platelet donations from 30 type O, 15 type A, and 15 type B donors collected using a Trima Accel Automated Blood Collection System were analyzed. Each donation provided two samples: donor whole blood retention samples (Retention) collected in EDTA tubes at the time of apheresis, and final platelet product post-CAD samples (Post-CAD) collected after processing.

All platelets were treated with the pathogen reduction INTERCEPT® Blood System for Platelets System (Cerus Corp.). In the manufacturing process, platelets are sterilely transferred into a single-use processing set containing amotosalen solution. The platelets are placed in an illumination device which delivers a controlled dose of ultraviolet A (UVA) light for each treatment, lasting approximately four minutes. After illumination, platelets are transferred to the bag containing the CAD and agitated for 6–24 h at room temperature. At completion of the CAD incubation, the platelets are transferred by gravity flow to the storage container in their final state as INTERCEPT platelets. The Post-CAD sample is then collected for testing.

Samples were tested for IgM and IgG classes of anti-A, anti-B, and anti-A/B isoagglutinins, totaling 360 individual tests split evenly between Retention and Post-CAD samples. ABO Isoagglutinin titers of both IgM and IgG classes were determined using solid-phase technology on the NEO Iris platform (Werfen, previously Immucor, Inc.) [14]. Initial IgM and IgG results were measured up to a dilution of 1:128. For IgG isoagglutinin titer results exceeding 128, reflex testing was conducted to establish titers up to a dilution of 1:2048. Automated protocols were unavailable for IgM titers above 128. In the event of invalid automation results, the test was repeated up to two times and excluded from the study upon the third invalid result.

In the automated IgM protocol, 50 µL of sample was serially diluted to a dilution of 1:128, then incubated with 15 µL of pooled A or B cells (2–4 %, Immucor) for 10 min at 20 °C. The IgG protocol utilizes Capture-R® technology to detect IgG red blood cell antibodies. Pooled A or B cells (2–4 %) were added to each well of Capture-R Select strips, followed by mixing with 50 µL of system fluid. After centrifugation and washing steps, 50 µL of system fluid and 100 µL of sample were added and serially diluted up to 1:128. Subsequently, 100 µL of Low Ionic Strength Saline (LISS) was added and incubated for 15 min at 39 °C, followed by washing. Capture-R® Indicator cells (55 µL) were added, and the plate was centrifuged, and read. Reflex testing employed the same IgG protocol at higher dilutions.

### Statistical analysis

Since ABO titers represent non-continuous data, the results were transformed into titer steps using a logarithmic (log_2_) transformation. Specifically, a titer of 0 corresponded to −1, a titer of 1 corresponded to 0, and so forth, with each doubling dilution represented by a single digit increase in titer steps. The differences in titer steps between Retention and Post-CAD samples were then assessed to demonstrate if one sample type produced higher or lower results. Meanwhile, the absolute difference was used to determine the percentage of concordance within ±1 and ±2 titer steps. To evaluate the statistical significance between Retention and Post-CAD IgM and IgG results, a non-parametric Wilcoxon paired signed-rank test (using Z distribution) was employed, with a significance level set at 0.05.

## Results

**Figure 1:**
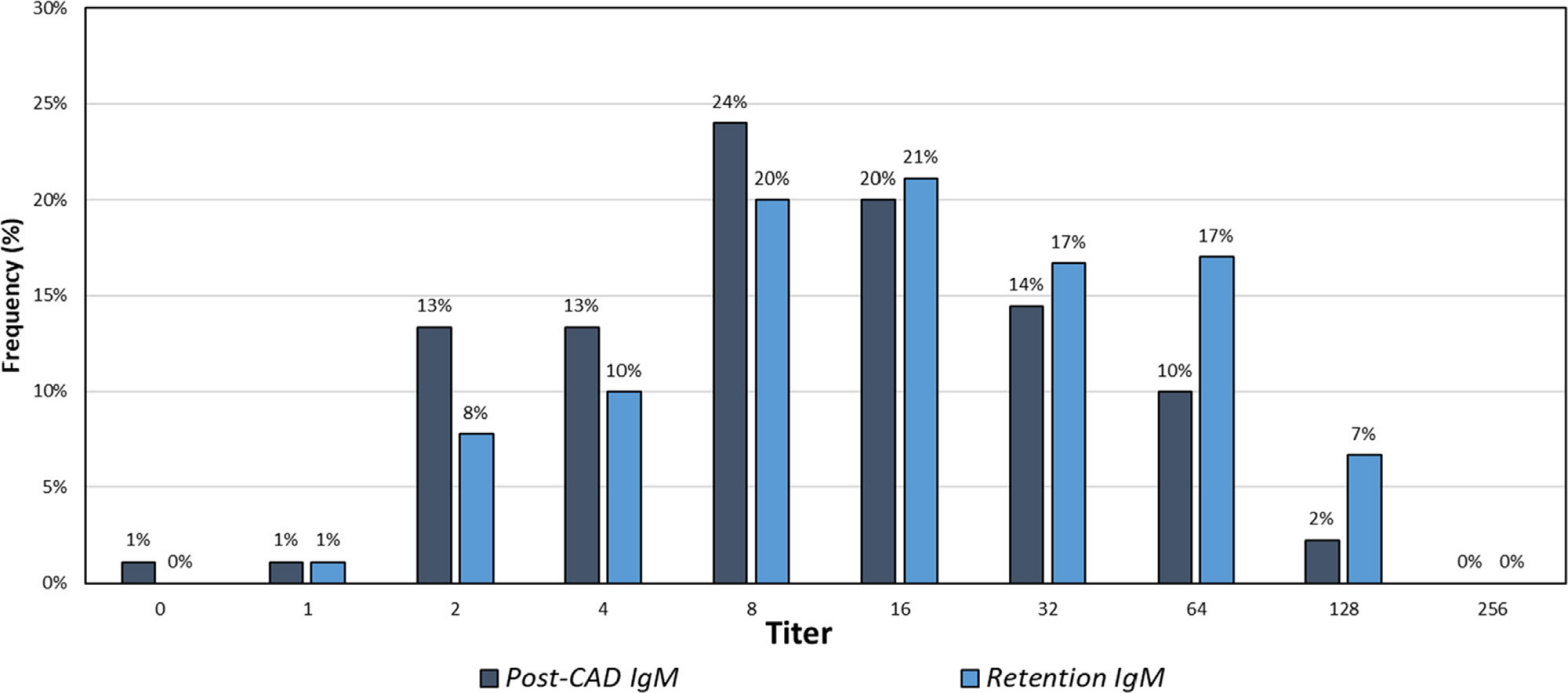
Distribution of ABO IgM isoagglutinin titers for retention and post-CAD samples.

The distribution of titer results for IgM class isoagglutinin titers was close to symmetrical (Figure 1): Post-CAD results had a median titer of 8, mode of 8, and a sample coefficient of variation (CV) of 0.50 (in titer steps), and the Retention results had a median titer of 16, mode of 16, and CV of 0.42.

**Figure 2:**
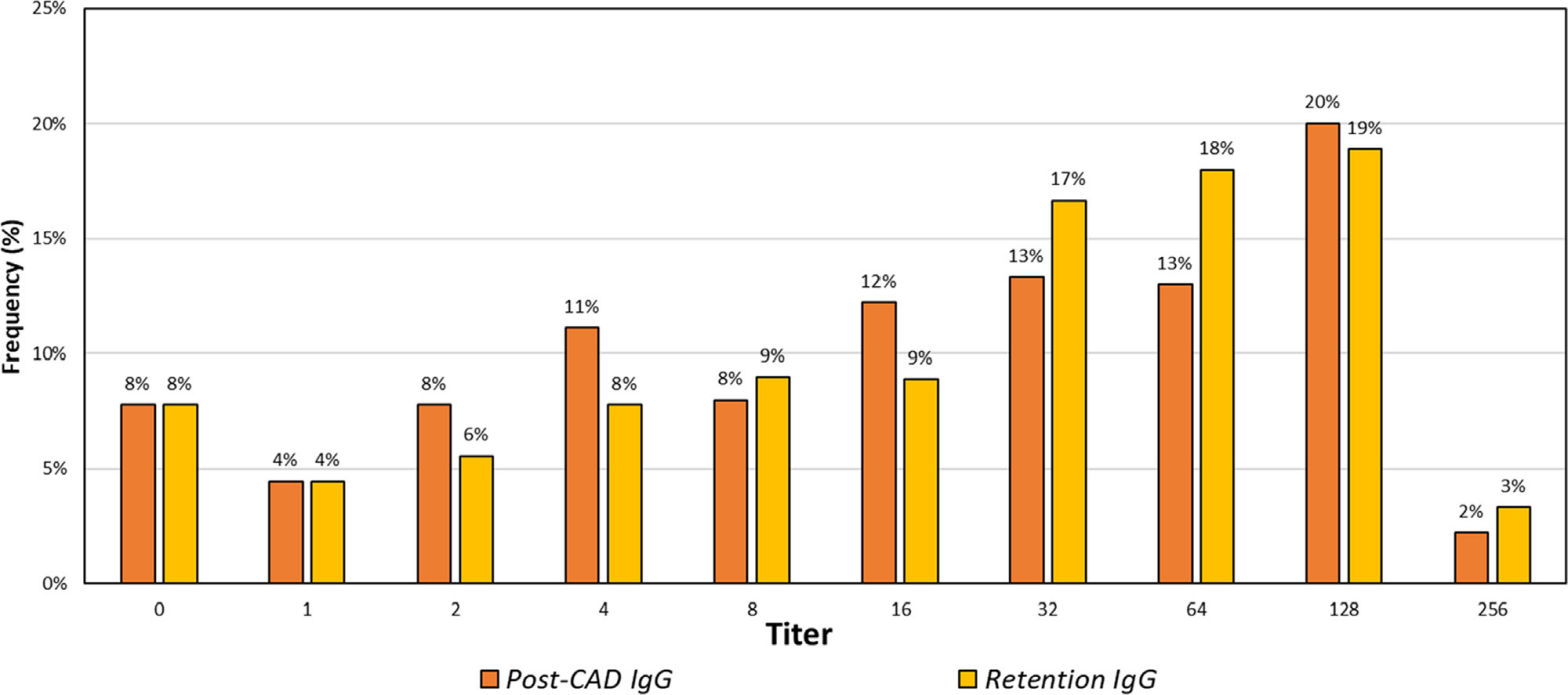
Distribution of ABO IgG isoagglutinin titers for retention and post-CAD samples.

The distribution for IgG class isoagglutinin titers (Figure 2) was asymmetrical: Post-CAD results had a median titer of 16, mode of 128, and CV of 0.65, and Retention results had a median of 32, mode of 128, and CV of 0.61. The data for IgG titers was more evenly distributed between titers 0 to 256, and skewed towards higher results.

**Figure 3:**
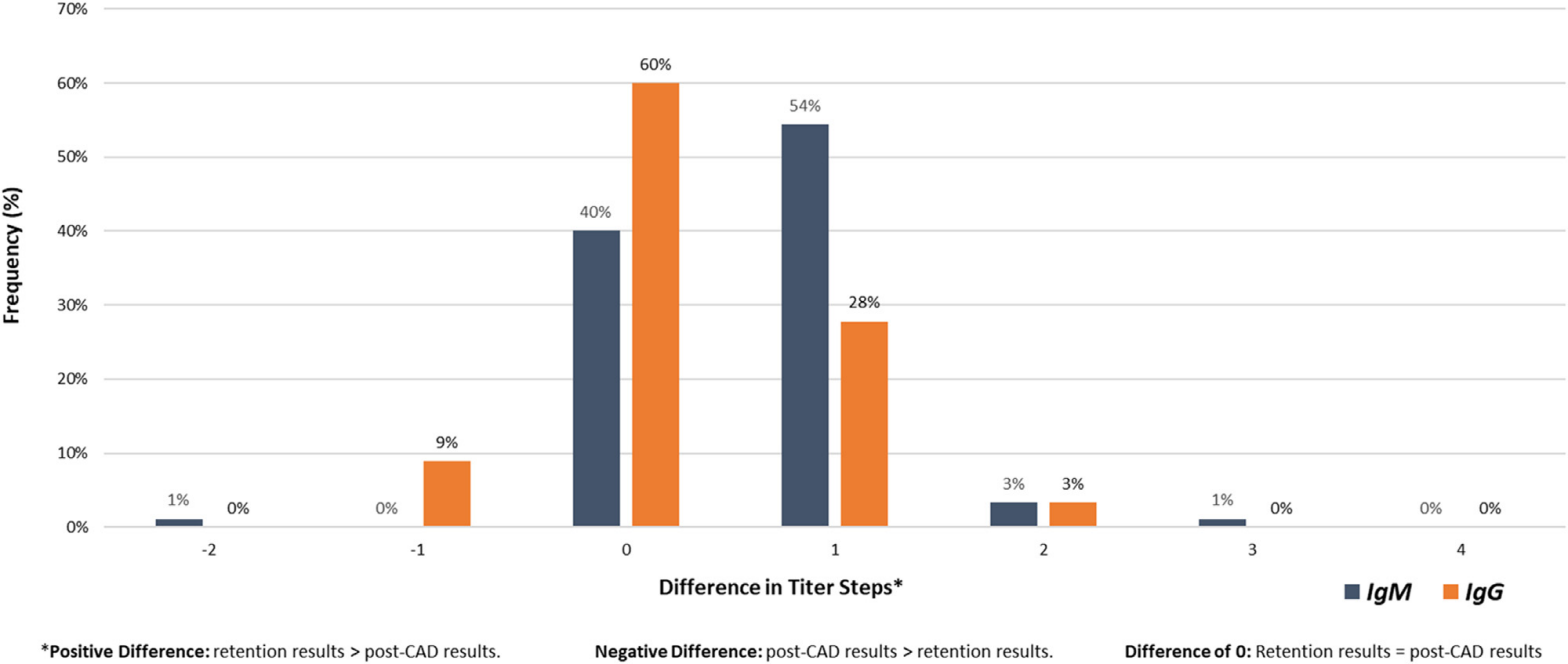
Distribution of titer step differences (%) (retention – Post-CAD).

Comparing IgM Post-CAD and Retention samples, 99 % of all Retention titer results were greater than or equal to their Post-CAD counterparts (Figure 3). An exception to this was a single outlier (1 %) where the Post-CAD IgM result was two titer steps greater than the corresponding Retention result. In contrast to IgM results, IgG data had 9 % of its Post-CAD samples yield higher titer results than the paired Retention samples.

Retention and Post-CAD samples were compared according to type and isoagglutinin class (IgM or IgG) using Wilcoxon paired signed-rank test. Overall, the p-values for both IgM and IgG titers were <0.05, indicating significant differences between the Post-CAD and Retention samples. Upon breaking down the antibody classes by blood type, the p-values for most comparisons indicated statistically significant differences between Post-CAD and Retention results (Table 1).

**Table 1:** Statistical results summary table.

Blood type	Sample n	Antibody specificity, class	Specimen source	Median	Mode	Min	Max	CV^a^	*p* value^b^	Concordance (%)^c^ +/−1	Concordance (%)^c^ +/−2
O	30	Anti-A, IgM	Retention	32	64	2	128	0.37	<0.05	93 %	100 %
			Post-CAD	16	64	2	64	0.41			
O	30	Anti-B, IgM	Retention	16	16	1	64	0.45	<0.05	100 %	100 %
			Post-CAD	8	8	0	64	0.49			
O	30	Anti-A, IgG	Retention	64	128	1	256	0.28	0.066	100 %	100 %
			Post-CAD	64	128	1	256	0.31			
O	30	Anti-B, IgG	Retention	32	128	2	128	0.36	<0.05	100 %	100 %
			Post-CAD	32	32	2	128	0.40			
A	15	Anti-B, IgM	Retention	8	8	2	128	0.50	<0.05	87 %	93 %
			Post-CAD	4	2	1	128	0.73			
A	15	Anti-B, IgG	Retention	1	0	0	8	3.32	0.484	100 %	100 %
			Post-CAD	1	0	0	8	2.50			
B	15^d^	Anti-A, IgM	Retention	16	32	2	128	0.38	0.095^d^	93 %	100 %
			Post-CAD	8	8	2	128	0.43			
B	15	Anti-A, IgG	Retention	8	32	0	64	0.60	<0.05	80 %	100 %
			Post-CAD	4	2	0	32	0.82			
All	60	Anti-A & Anti-B, IgM	Retention	16	16	1	128	0.42	<0.05	94 %	99 %
			Post-CAD	8	8	0	128	0.50			
All	60	Anti-A & Anti-B, IgG	Retention	32	128	0	256	0.65	<0.05	97 %	100 %
			Post-CAD	16	128	0	256	0.61			

^a^ Coefficient of variation was calculated using titer steps.

^b^ The results were compared using non-parametric Wilcoxon signed ranked test (using Z distribution) with a significance level at 0.05.

^c^ Concordance is calculated using absolute difference in titers steps between retention and post-CAD samples.

^d^ One of 15 Type B, IgM samples was an outlier with a post-CAD result two titer steps above its corresponding retention sample. This was the only pair of samples out of 60 pairs that had a higher IgM titer on the post-CAD sample than the retention sample. When the outlier is excluded (*n* = 14, for type B) from the analysis the *p* value is below 0.05 (*p* value of 0.006).

There were three cases in which the p-values exceeded 0.05: anti-A IgG for group O, anti-B IgG for group A, and anti-A IgM for group B. In the last case, the high p-value was attributed to a single outlier which, when excluded (*n* = 14 for type B) from the statistical test, yielded a p-value of less than 0.05 (*p*-value = 0.006). The percentage concordance between Retention and Post-CAD samples was calculated using titer steps. All comparisons had 100 % concordance within ±2 titer steps.

**Figure 4:**
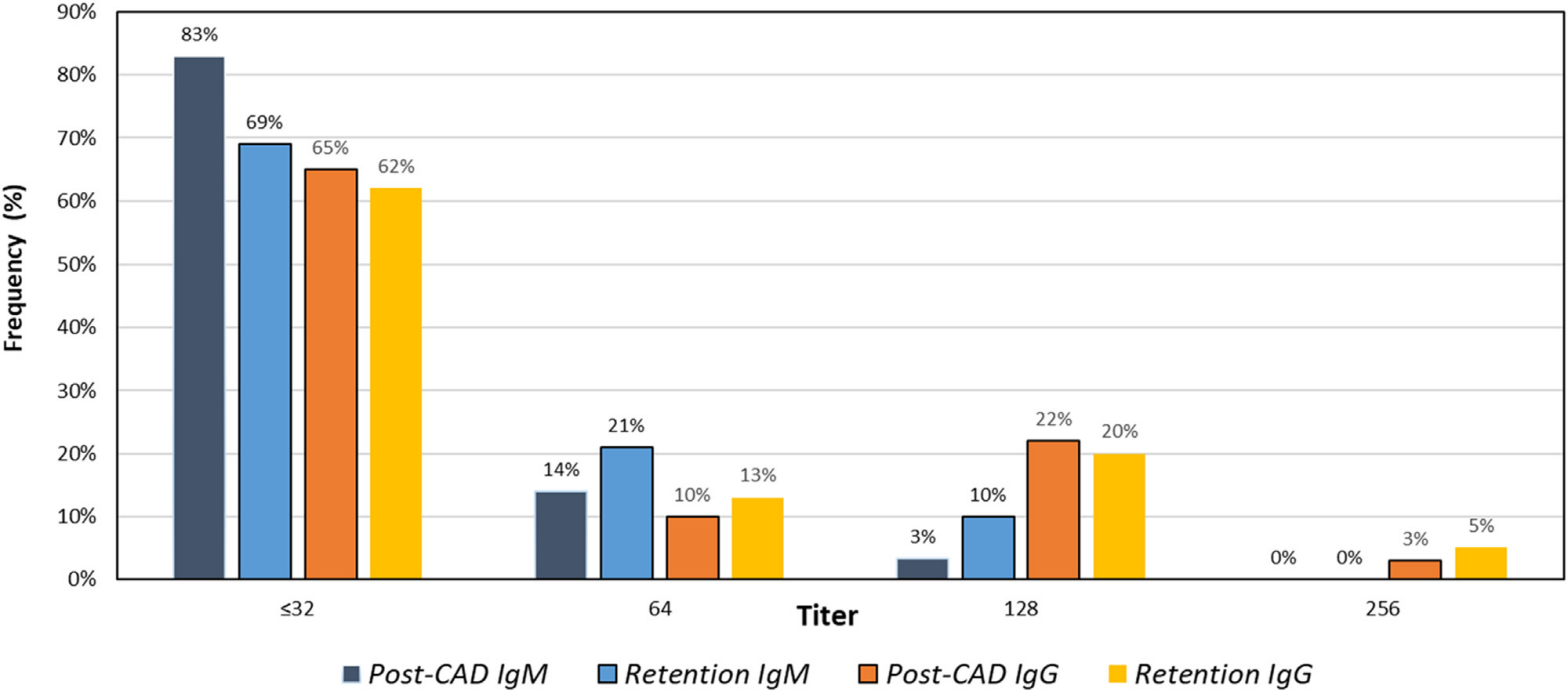
Distribution of maximum titer results (anti-A or anti-B) per sample tested.

Distribution of titers from all blood group results utilizing maximum titer per sample was determined (Figure 4). Since blood type O contains antibodies of both anti-A and anti-B specificity, the higher of the two results was used to determine the maximum titer. For IgM, 83 % of all Post-CAD samples and 69 % of all Retention samples yielded titer results ≤32. For IgG, 65 % of Post-CAD and 62 % of Retention samples yielded titer results ≤32.

The distribution of the differences between IgG and IgM (IgG-IgM) results was similar when comparing Post-CAD and Retention samples. However, in both cases IgG isoagglutinin testing generally yielded higher titer results than the IgM counterparts. Refer to Table 1 to compare median titer results between IgM and IgG class isoagglutinin titers.

## Discussion

The ABO blood group system plays a pivotal role in transfusion medicine, dictating the compatibility between donor and recipient blood types [15]. In vulnerable patient populations such as pediatric patients, ensuring compatibility is essential to mitigating the risk of adverse reactions during platelet transfusions [16]. This study investigated the quantitative assessment of ABO isoagglutinin titers, focusing on both IgM and IgG classes, in platelet donations using solid-phase technology [17]. By comparing titers between donor Retention samples and final platelet Post-CAD samples, this study aimed to evaluate the impact of the pathogen reduction manufacturing process on isoagglutinin levels.

The results revealed notable differences in the distribution of isoagglutinin titers between Retention and Post-CAD samples. Specifically, the median and mode titers for both IgM and IgG classes differed between the two sample types. For IgM isoagglutinin titers, Retention samples exhibited higher median and mode titers compared to Post-CAD samples. These differences suggest that the manufacturing process influences isoagglutinin levels in platelet products. Specifically, 99 % of IgM Retention titers were greater than or equal to their Post-CAD counterparts, with only a single outlier. In contrast, IgG results showed a more variable pattern, with 9 % of Post-CAD samples exhibiting higher titers than Retention samples, and a greater percentage of equivalent results between Retention and Post-CAD titers compared to IgM.

The observed reduction in isoagglutinin titers in Post-CAD samples can be attributed to the pathogen reduction process using the INTERCEPT® Blood System, which includes psoralen treatment and UV light activation [18]. This process not only inactivates pathogens but may also impact the isoagglutinin levels by binding and possibly denaturing these antibodies, thereby reducing their effective concentration in the final platelet product. This reduction is beneficial in minimizing the risk of hemolytic transfusion reactions, particularly in pediatric patients who are more susceptible to such adverse events [19].

The statistical analysis, using the Wilcoxon paired signed-rank test, indicated significant differences between Retention and Post-CAD samples for both IgM and IgG titers, with *p*-values <0.05 in most comparisons. This finding underscores the consistent impact of the manufacturing process on reducing isoagglutinin titers across different blood types and antibody classes.

The percentage concordance within ±2 titer steps was 100 % for all comparisons, indicating a high degree of consistency between Retention and Post-CAD samples, even with the observed reductions in isoagglutinin titers. This high concordance rate supports the reliability of the pathogen reduction process in maintaining relative titer levels while reducing absolute concentrations.

Notably, three comparisons did not reach statistical significance: anti-A IgG for group O, anti-B IgG for group A, and anti-A IgM for group B. The high p-value for anti-B IgG for group A is likely due to the predominance of negative results (mode of 0), which diminishes the potential for detecting significant differences. After exclusion of the outlier in the anti-A IgM for group B, the p-value reached statistical significance. Anti-A IgG for group O had a p-value of 0.066, where a minor increase in the sample size could make it significant.

The reduction in isoagglutinin titers after pathogen reduction highlights the potential for safer out-of-group platelet transfusions with pathogen-reduced platelets, particularly in settings where ABO-matched platelets are scarce [20]. By lowering the risk of hemolytic reactions, pathogen-reduced platelets can be more safely used across different patient populations, including vulnerable groups such as pediatric patients. This aligns with current trends in transfusion medicine that emphasize both safety and availability of blood products [21]. Moreover, the use of solid-phase technology on the NEO Iris platform for titer measurement offers a robust and standardized approach for assessing isoagglutinin levels [22]. This technological advancement facilitates high-throughput, automated testing, enhancing the efficiency and accuracy of compatibility assessments in transfusion services.

While this study provides significant insights, it has limitations that should be addressed in future research. The sample size, although adequate for demonstrating significant differences, could be expanded to include a more diverse range of donor demographics. Additionally, the impact of other variables such as storage duration and donor health status on isoagglutinin titers could be further investigated.

In conclusion, this study highlights the significant reduction in ABO isoagglutinin titers achieved through the pathogen reduction process, enhancing the safety profile of out-of-group platelet transfusions. The use of advanced solid-phase technology for titer assessment ensures precise and reliable measurements, supporting informed clinical decision-making [23]. These findings contribute to the evolving landscape of transfusion medicine, promoting safer and more effective blood product utilization.

## Contribution of the author

Mikayel Yeghiazaryan: Contributed to data review, data cleaning, figure generation and supported statistical interpretation.

Yembur Ahmad: Assisted in data analysis and critically revised the manuscript for important intellectual content.

Jessie Singer: Performed literature review, organized and extract data, and contributed to manuscript writing.

Vaanush Nazaryan: Participated in data management and quality control, and supported results interpretation.

Craig Fletcher: Critically revised the manuscript for important intellectual content.

Yamac Akgun: Conceived and designed the study, conducted statistical analysis, interpreted results, supervised the research process, and reviewed the manuscript for final approval.

## Conflicts of interest

The authors declare no conflicts of interest.

## References

[1] Moncharmont P. (2018). Platelet component transfusion and alloimmunization: where do we stand?. Transfus Clin Biol.

[2] Dunbar N.M. (2020). Does ABO and RhD matching matter for platelet transfusion?. Hematol Am Soc Hematol Educ Program.

[3] Filitti-Wurmser S. (1976). Natural antibodies and immune antibodies of human ABO blood group system. Biochimie.

[4] Girelli G., Antoncecchi S., Casadei A.M., Del Vecchio A., Isernia P., Motta M. (2015). Recommendations for transfusion therapy in neonatology. Blood Transfus.

[5] Blumberg N., Refaai M., Heal J. (2015). ABO matching of platelet transfusions - "start making sense". "As we get older, and stop making sense…" - the Talking Heads (1984). Blood Transfus.

[6] Han M.H., Badami K.G. (2024). ABO non-identical platelet transfusions, immune platelet refractoriness and platelet support. Br J Haematol.

[7] Kaufman R.M., Djulbegovic B., Gernsheimer T., Kleinman S., Tinmouth A.T., Capocelli K.E., AABB (2015). Platelet transfusion: a clinical practice guideline from the AABB. Ann Intern Med.

[8] Shirey R.S., Cai W., Montgomery R.A., Chhibber V., Ness P.M., King K.E. (2010). Streamlining ABO antibody titrations for monitoring ABO-incompatible kidney transplants. Transfusion.

[9] Berséus O., Boman K., Nessen S.C., Westerberg L.A. (2013). Risks of hemolysis due to anti-A and anti-B caused by the transfusion of blood or blood components containing ABO-incompatible plasma. Transfusion.

[10] Nayak S., Makroo R.N., Prakash B., Chandra T., Agrawal S., Chowdhry M., Mohapatra A. (2019). Comparative evaluation of five different methods of anti-ABO antibody titration: an aid for ABO-incompatible organ transplants. Ther Apher Dial.

[11] Magron A., Laugier J., Provost P., Boilard E. (2018). Pathogen reduction technologies: the pros and cons for platelet transfusion. Platelets.

[12] Pelletier J.P., Transue S., Snyder E.L. (2006). Pathogen inactivation techniques. Best Pract Res Clin Haematol.

[13] Ciaravi V., McCullough T., Dayan A.D. (2001). Pharmacokinetic and toxicology assessment of intercept (S-59 and UVA treated) platelets. Hum Exp Toxicol.

[14] Inc Immucor (2019).

[15] Cooling L. (2007). ABO and platelet transfusion therapy. Immunohematology.

[16] Patel R.M., Josephson C. (2019). Neonatal and pediatric platelet transfusions: current concepts and controversies. Curr Opin Hematol.

[17] Lally K., Kruse R.L., Smetana H., Davis R., Roots A., Marshall C. (2020). Isohemagglutinin titering performed on an automated solid-phase and hemagglutinin-based analyzer is comparable to results obtained by manual gel testing. Transfusion.

[18] Osman A., Hitzler W.E., Provost P. (2016). Peculiarities of studying the effects of pathogen reduction technologies on platelets. Proteomics Clin Appl.

[19] Harris S.B., Josephson C.D., Kost C.B., Hillyer C.D. (2007). Nonfatal intravascular hemolysis in a pediatric patient after transfusion of a platelet unit with high-titer anti-A. Transfusion.

[20] Dunbar N.M., Ornstein D.L., Dumont L.J. (2012). ABO incompatible platelets: risks versus benefit. Curr Opin Hematol.

[21] Blumberg N., Heal J.M. (2023). How do we forecast tomorrow's transfusion? - next generation transfusion practices to improve recipient safety. Transfus Clin Biol.

[22] Niu S., Vetsch M., Beaudin L., Bodnar M., Clarke G. (2023). Comparison of automated solid phase versus manual saline indirect antiglobulin test methodology for non-ABO antibody titration: implications for perinatal antibody monitoring. Transfusion.

[23] Schneider D., Vicarioto M., Coluzzi S., Matteocci A., Revelli N., Foglieni B. (2022). ABO antibody titres: a multisite comparative study of equivalency and reproducibility for automated solid-phase and haemagglutination titration, and manual dilution with gel column agglutination technology. Blood Transfus.

